# *N*,*N*′-[(8-*endo*,11-*endo*-Dihy­droxy­penta­cyclo­[5.4.0.0^2,6^.0^3,10^.0^5,9^]undecane-8,11-di­yl)bis­(methyl­enecarbon­yl)]di-l-phenyl­alanine

**DOI:** 10.1107/S1600536810035956

**Published:** 2010-09-11

**Authors:** Rajshekhar Karpoormath, Patrick Govender, Hendrik G. Kruger, Thavendran Govender, Glenn E. M. Maguire

**Affiliations:** a School of Chemistry, University of KwaZulu–Natal, Durban 4000, South Africa; b Department of Biochemistry, University of KwaZulu–Natal, Durban 4000, South Africa; c School of Pharmacy and Pharmacology, University of KwaZulu–Natal, Durban 4000, South Africa

## Abstract

The title compound, C_33_H_36_N_2_O_8_, is the first example of a disubstituted peptidic pentacycloundecane (PCU) diol. The structure displays an array of inter- and intra­molecular hydrogen bonding by both amide and alcohol functional groups. This hydrogen-bonding system connects the mol­ecules into a three-dimensional network.

## Related literature

For examples of PCU cage structures which exhibit C—C bond lengths that deviate from the norm, see: Flippen-Anderson *et al.* (1991 ▶); Linden *et al.* (2005 ▶); Kruger *et al.* (2005 ▶, 2006 ▶). For analogous PCU cage structures and their packing, see: Kruger *et al. (*2006); Boyle *et al.* (2007*a* ▶,*b* ▶); Vasquez *et al.* (2002 ▶); Anderson *et al.* (2007 ▶). For different cage crystal structures, see: Bott *et al.* (1998 ▶).
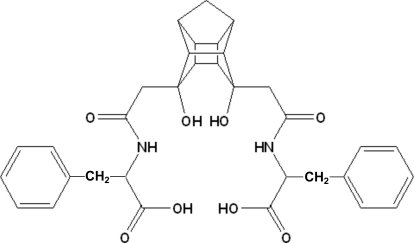


## Experimental

### 

#### Crystal data

C_33_H_36_N_2_O_8_
*M* *_r_* = 588.64
Orthorhombic, 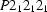

*a* = 10.6230 (5) Å
*b* = 14.7773 (6) Å
*c* = 18.2819 (8) Å
*V* = 2869.9 (2) Å^3^
*Z* = 4
Cu *K*α radiation
μ = 0.80 mm^−1^
*T* = 100 K
0.33 × 0.28 × 0.18 mm

#### Data collection

Bruker Kappa DUO APEXII diffractometer
Absorption correction: multi-scan (*SADABS*; Sheldrick, 1997 ▶) *T*_min_ = 0.678, *T*_max_ = 0.875
17910 measured reflections
4968 independent reflections
4892 reflections with *I* > 2σ(*I*)
*R*_int_ = 0.018

#### Refinement

*R*[*F* ^2^ > 2σ(*F* ^2^)] = 0.032
*wR*(*F* ^2^) = 0.085
*S* = 1.06
4968 reflections
412 parameters
6 restraints
H atoms treated by a mixture of independent and constrained refinement
Δρ_max_ = 0.63 e Å^−3^
Δρ_min_ = −0.19 e Å^−3^
Absolute structure: Flack (1983 ▶), 2058 Friedel pairs
Flack parameter: −0.02 (14)

### 

Data collection: *APEX2* (Bruker, 2006 ▶); cell refinement: *SAINT* (Bruker, 2006 ▶); data reduction: *SAINT*; program(s) used to solve structure: *SHELXS97* (Sheldrick, 2008 ▶); program(s) used to refine structure: *SHELXL97* (Sheldrick, 2008 ▶); molecular graphics: *X-SEED* (Barbour, 2001 ▶); software used to prepare material for publication: *SHELXL97*.

## Supplementary Material

Crystal structure: contains datablocks I, global. DOI: 10.1107/S1600536810035956/gw2085sup1.cif

Structure factors: contains datablocks I. DOI: 10.1107/S1600536810035956/gw2085Isup2.hkl

Additional supplementary materials:  crystallographic information; 3D view; checkCIF report

## Figures and Tables

**Table 1:** Hydrogen-bond geometry (Å, °)

*D*—H⋯*A*	*D*—H	H⋯*A*	*D*⋯*A*	*D*—H⋯*A*
N1—H1*N*⋯O6^i^	0.95 (1)	1.90 (1)	2.8399 (18)	172 (2)
N2—H2*N*⋯O5	0.96 (1)	2.01 (2)	2.7579 (18)	134 (2)
O1—H1*O*⋯O2	0.96 (1)	1.79 (2)	2.6649 (16)	150 (2)
O5—H5*O*⋯O1	0.95 (1)	1.58 (1)	2.4886 (16)	158 (3)
O4—H4*O*⋯O7^ii^	0.96 (1)	1.84 (2)	2.7425 (18)	154 (2)
O8—H8*O*⋯O5^iii^	0.97 (1)	1.68 (1)	2.6501 (17)	177 (3)

Symmetry codes: (i) 
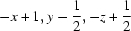
; (ii) 
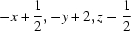
; (iii) 
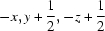
.

**Fig. 1.**
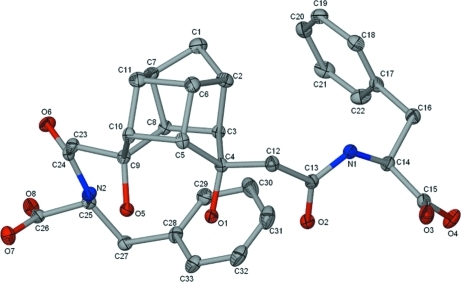
Molecular structure of (1) showing numbering scheme with all hydrogen atoms omitted for clarity. All non-hydrogen atoms are shown as ellipsoids with probability level of 40%.

**Fig. 2.**
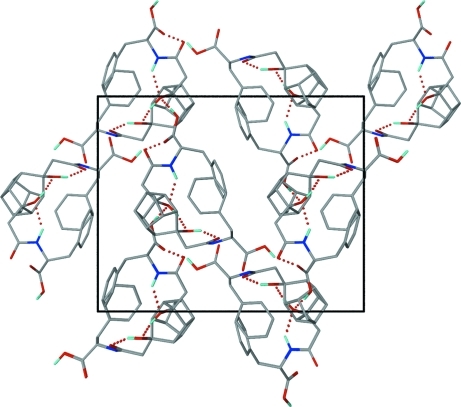
Projection viewed along [100]. Only the hydrogen atoms involved in hydrogen bonds are shown. Other hydrogen atoms are omitted for clarity. The hydrogen bonds are shown as dotted lines.

## supplementary crystallographic information

### Comment

The novel compound (I) was synthesized as a part of an ongoing project looking into the biological activity of cage compounds and their derivatives. It can be converted to a diacid and coupled to desired peptides as a potential HIV-1 protease inhibitor. The title compound (I) consists of a large apolar (lipophilic) hydrocarbon skeleton with polar amide and hydroxy units (Fig.1). (I) crystallized with four molecules in the asymmetric unit all of which show shortening and elongation of specific C—C bonds in the cage moiety as observed by previous authors (Flippen-Anderson *et al.*, 1991; Linden *et al.*, 2005; Kruger *et al.*, 2005; Kruger *et al.*, 2006, Boyle *et al.*, 2007a,b). The shortest C—C bond lengths in the cage occur between C1—C7, C1—C2, C4—C5 and C9—C10, with the values ranging between 1.499–1.533 Å. The longest C—C bond length is between C6—C11 with a value of 1.616 (3) Å. This is the first example of a bis-amino acid substituted pentacyloundecane diol reported. We believe it to be the primary example of a PCU diol with aromatic residues positioned close to the cage. As the phenylalanine derivative it is interesting to see that there are no obvious π-stacking contributions to the overall structure. A striking aspect of the structure is its hydrogen bonding arrangements. In previous examples PCU diols were reported as being hydrogen bonded in both intra and intermolecular fashion giving rise to two-dimensional crystal planes (Vasquez *et al.*, 2002). In (I) there are several possibly sites for hydrogen bonding to occur and all of the centers do in fact take an active part. The hydrogen bonding arrangements can be seen in Fig. 2. Intramolecular hydrogen bonding occurs between the amide N(2)–H···O5, the alcohol group O(5)–H···O(1) and the next alcohol O(1)–H···O(2). This is a similar arrangement to that found by Anderson *et al.*, 2007 when three and four alcohol groups respectively were reported. In the case of intermolecular bonding a far more intricate arrangement occurs than previously reported examples. An intermolecular hydrogen bond is generated by the amide group N(1)–H···O(6) (symmetry code; -*x* + 1, *y* - 1/2, -*z* + 1/2). All of the other intermolecular bonds are formed by the carboxylic residues in two distinct arrangements. First, by O(8)–H···O(5) on the PCU cage (symmetry code; -*x*, *y* + 1/2, -*z* + 1/2). Second by O(4)–H···O(7) at the terminals of the two molecules (symmetry code; -*x* + 1/2, -*y* + 2, *z* - 1/2). These interactions create the interlocking arrangements rendering the three dimensional expansion of the structure.

### Experimental

A solution of PCU cage diol diacid (0.50 g, 1.7 mmol) in dry DCM (15 ml) was stirred at room temperature for 5 min. To this mixture was added *tert*-butyl 2-amino-3-phenylpropanoate (1.50 g, 6.8 mmol) and cooled in ice water bath and stirred for 5 min. To the above cooled mixture was added HATU (3.24 g, 8.5 mmol) followed by DIPEA (2.4 ml, 13.6 mmol) as a base. The solution was then slowly brought to room temperature and stirred for 6 h. The crude reaction mixtures was washed with water (100 ml) and then with 10% HCL (100 ml). The organic layer was dried over anhydrous sodium sulfate (Na_2_SO_4_) and filtered. The crude product was evaporated to dryness under vacuum using a teflon pump at 40 °C to obtain thick yellow oil. This crude oily product was further dissolved in DCM and TFA (1:1) solvent mixture and stirred overnight. TFA was removed by bubbling air through the peptide and the remaining DCM was removed under vacuum at 30°C. The product was obtained as a yellow oil which was purified by preparative HPLC and solid phase extraction. Crystallization of the product was carried out by dissolving the pure compound in DCM and TFA (1:1, 3 ml) and was stored at 20 °C. The percentage yield of the pure final compound was 67% (0.67 g).

1H NMR (MeOD, 600 MHz): δH 1.05 (1*H*, d, *J* = 10.68), 1.39 (1*H*, d, *J* = 10.56), 1.67 (1*H*, d, *J* = 8.84 Hz), 1.90 (1*H*, d, *J* = 8.88 Hz), 2.20–2.36 (7*H*, m), 2.45–2.46 (2*H*, m), 2.91–2.99 (2*H*, m), 3.24–3.29 (2*H*, m), 4.69–4.71 (1*H*, m), 4.77–4.87 (1*H*, m), 7.13–7.16 (1*H*, m), 7.21–7.30 (9*H*, m).

IR (film) v_max_: 3261.55 cm^-1^, 2957.06 cm^-1^, 1758.52 cm^-1^, 1638.17 cm^-1^, 1522.71 cm^-1^, 1191.53 cm^-1^, 758.74 cm^-1^, 701.36 cm^-1^, 489.06 cm^-1^.

Melting point: 414–415 K. HR ESI m/*z*: Calc. for C33H36N2O8: [*M*+H]+ m/*z* 589.2544. Found: [*M*+H]+ m/*z* 589.2541.

### Refinement

The crystal structure was solved by direct methods using *SHELXS* (Sheldrick, 2008). Non-hydrogen atoms were first refined isotropically followed by anisotropic refinement by full matrix least-squares calculations based on F^2^ using *SHELXL* (Sheldrick, 2008) using the graphics interface *X-SEED* (Barbour, 2001). Hydrogen atoms, first located in the difference map, were positioned geometrically and allowed to ride on their respective parent atoms, with C—H bond lengths of 1.00 (CH), 0.99 (CH_2_), or 0.98 (CH_3_). They were then refined with a riding model with *U*_iso_(H) = 1.5*U*_eq_(CH_3_) and *U*_iso_(H) = 1.2*U*_eq_(*X*) for *X* = CH or CH_2_.

### Crystal data

**Table:**

C_33_H_36_N_2_O_8_	*D*_x_ = 1.362 Mg m^−^^3^
*M**_r_* = 588.64	Melting point: 414 K
Orthorhombic, *P*2_1_2_1_2_1_	Cu *K*α radiation, λ = 1.54199 Å
Hall symbol: P 2ac 2ab	Cell parameters from 4968 reflections
*a* = 10.6230 (5) Å	θ = 3.9–67.3°
*b* = 14.7773 (6) Å	µ = 0.80 mm^−^^1^
*c* = 18.2819 (8) Å	*T* = 100 K
*V* = 2869.9 (2) Å^3^	Needle, colourless
*Z* = 4	0.33 × 0.28 × 0.18 mm
*F*(000) = 1248	

### Data collection

**Table:**

Bruker Kappa DUO APEXII diffractometer	4968 independent reflections
Radiation source: fine-focus sealed tube	4892 reflections with *I* > 2σ(*I*)
graphite	*R*_int_ = 0.018
1.2° φ scans and ω scans	θ_max_ = 67.3°, θ_min_ = 3.9°
Absorption correction: multi-scan (*SADABS*; Sheldrick, 1997)	*h* = −12→12
*T*_min_ = 0.678, *T*_max_ = 0.875	*k* = −17→17
17910 measured reflections	*l* = −21→21

### Refinement

**Table:**

Refinement on *F*^2^	Secondary atom site location: difference Fourier map
Least-squares matrix: full	Hydrogen site location: inferred from neighbouring sites
*R*[*F*^2^ > 2σ(*F*^2^)] = 0.032	H atoms treated by a mixture of independent and constrained refinement
*wR*(*F*^2^) = 0.085	*w* = 1/[σ^2^(*F*_o_^2^) + (0.0484*P*)^2^ + 0.8276*P*] where *P* = (*F*_o_^2^ + 2*F*_c_^2^)/3
*S* = 1.06	(Δ/σ)_max_ < 0.001
4968 reflections	Δρ_max_ = 0.63 e Å^−^^3^
412 parameters	Δρ_min_ = −0.19 e Å^−^^3^
6 restraints	Absolute structure: Flack (1983), 2058 Friedel pairs
Primary atom site location: structure-invariant direct methods	Flack parameter: −0.02 (14)

### Special details

**Table:**

Experimental. Half sphere of data collected using *COLLECT* strategy (Nonius, 2000). Crystal to detector distance = 45 mm; combination of φ and ω scans of 1.2°, 80 s per °, 2 iterations.
Geometry. All e.s.d.'s (except the e.s.d. in the dihedral angle between two l.s. planes) are estimated using the full covariance matrix. The cell e.s.d.'s are taken into account individually in the estimation of e.s.d.'s in distances, angles and torsion angles; correlations between e.s.d.'s in cell parameters are only used when they are defined by crystal symmetry. An approximate (isotropic) treatment of cell e.s.d.'s is used for estimating e.s.d.'s involving l.s. planes.
Refinement. Refinement of *F*^2^ against ALL reflections. The weighted *R*-factor *wR* and goodness of fit *S* are based on *F*^2^, conventional *R*-factors *R* are based on *F*, with *F* set to zero for negative *F*^2^. The threshold expression of *F*^2^ > σ(*F*^2^) is used only for calculating *R*-factors(gt) *etc*. and is not relevant to the choice of reflections for refinement. *R*-factors based on *F*^2^ are statistically about twice as large as those based on *F*, and *R*- factors based on ALL data will be even larger.

### Fractional atomic coordinates and isotropic or equivalent isotropic displacement parameters (Å^2^)

**Table:**

	*x*	*y*	*z*	*U*_iso_*/*U*_eq_	
O1	0.26914 (11)	0.87395 (8)	0.16405 (6)	0.0244 (2)	
H1O	0.299 (2)	0.8757 (16)	0.1144 (7)	0.044 (6)*	
O2	0.40843 (12)	0.84037 (9)	0.04589 (7)	0.0301 (3)	
O3	0.60107 (13)	0.69337 (9)	−0.04532 (7)	0.0347 (3)	
O4	0.64015 (15)	0.80350 (9)	−0.12717 (7)	0.0377 (3)	
H4O	0.620 (3)	0.7565 (13)	−0.1617 (12)	0.057 (7)*	
O5	0.15544 (11)	0.99230 (8)	0.23485 (6)	0.0230 (2)	
H5O	0.181 (2)	0.9478 (13)	0.2006 (12)	0.053 (7)*	
O6	0.21685 (12)	1.25096 (8)	0.32318 (7)	0.0291 (3)	
O7	−0.06852 (12)	1.28739 (9)	0.24885 (7)	0.0317 (3)	
O8	0.03196 (12)	1.40531 (8)	0.20026 (7)	0.0314 (3)	
H8O	−0.035 (2)	1.4387 (18)	0.2239 (15)	0.072 (9)*	
N1	0.61453 (14)	0.81718 (10)	0.06881 (7)	0.0241 (3)	
H1N	0.6770 (17)	0.7959 (15)	0.1017 (11)	0.042 (6)*	
N2	0.13816 (14)	1.17823 (9)	0.22652 (7)	0.0237 (3)	
H2N	0.120 (2)	1.1205 (10)	0.2053 (13)	0.046 (6)*	
C1	0.60049 (19)	1.03551 (14)	0.28800 (11)	0.0371 (4)	
H1A	0.6632	1.0307	0.3279	0.045*	
H1B	0.6278	1.0809	0.2515	0.045*	
C2	0.56791 (17)	0.94515 (12)	0.25429 (10)	0.0298 (4)	
H2	0.6403	0.9140	0.2300	0.036*	
C3	0.45567 (16)	0.96466 (11)	0.20319 (9)	0.0214 (3)	
H3	0.4825	0.9846	0.1533	0.026*	
C4	0.38679 (15)	0.87291 (11)	0.20250 (9)	0.0220 (3)	
C5	0.37308 (16)	0.85663 (11)	0.28471 (9)	0.0248 (3)	
H5	0.3493	0.7934	0.2986	0.030*	
C6	0.50323 (18)	0.88894 (13)	0.31455 (10)	0.0320 (4)	
H6	0.5556	0.8445	0.3424	0.038*	
C7	0.46939 (18)	1.05156 (13)	0.31537 (10)	0.0307 (4)	
H7	0.4585	1.1100	0.3422	0.037*	
C8	0.38393 (16)	1.04175 (11)	0.24716 (9)	0.0218 (3)	
H8	0.3765	1.0993	0.2187	0.026*	
C9	0.25780 (16)	1.01308 (10)	0.28335 (9)	0.0216 (3)	
C10	0.30414 (16)	0.93324 (11)	0.32961 (9)	0.0232 (3)	
H10	0.2433	0.9112	0.3674	0.028*	
C11	0.43220 (18)	0.96760 (13)	0.36003 (10)	0.0314 (4)	
H11	0.4439	0.9695	0.4143	0.038*	
C12	0.46489 (16)	0.79656 (11)	0.16772 (9)	0.0256 (4)	
H12A	0.5445	0.7888	0.1952	0.031*	
H12B	0.4174	0.7390	0.1704	0.031*	
C13	0.49385 (16)	0.81869 (11)	0.08861 (9)	0.0236 (3)	
C14	0.65210 (17)	0.84505 (12)	−0.00387 (9)	0.0257 (4)	
H14	0.5983	0.8978	−0.0179	0.031*	
C15	0.62849 (17)	0.77022 (12)	−0.05975 (10)	0.0276 (4)	
C16	0.78958 (17)	0.87780 (12)	−0.00307 (10)	0.0289 (4)	
H16A	0.8445	0.8295	0.0167	0.035*	
H16B	0.8172	0.8913	−0.0536	0.035*	
C17	0.80165 (16)	0.96194 (12)	0.04362 (10)	0.0265 (4)	
C18	0.85097 (17)	0.95746 (12)	0.11397 (10)	0.0300 (4)	
H18	0.8838	0.9018	0.1316	0.036*	
C19	0.85280 (18)	1.03328 (13)	0.15874 (11)	0.0323 (4)	
H19	0.8856	1.0290	0.2070	0.039*	
C20	0.80709 (18)	1.11473 (13)	0.13338 (11)	0.0328 (4)	
H20	0.8080	1.1665	0.1641	0.039*	
C21	0.76010 (19)	1.12083 (13)	0.06335 (12)	0.0372 (4)	
H21	0.7293	1.1770	0.0455	0.045*	
C22	0.7578 (2)	1.04459 (13)	0.01874 (11)	0.0357 (4)	
H22	0.7256	1.0493	−0.0296	0.043*	
C23	0.20364 (16)	1.08852 (11)	0.33230 (9)	0.0243 (3)	
H23A	0.1209	1.0686	0.3513	0.029*	
H23B	0.2603	1.0970	0.3747	0.029*	
C24	0.18689 (15)	1.17912 (11)	0.29397 (9)	0.0219 (3)	
C25	0.12644 (16)	1.26173 (11)	0.18574 (9)	0.0235 (3)	
H25	0.2069	1.2964	0.1899	0.028*	
C26	0.01960 (16)	1.31895 (11)	0.21642 (9)	0.0235 (3)	
C27	0.09994 (16)	1.24235 (12)	0.10448 (9)	0.0262 (4)	
H27A	0.0140	1.2172	0.0995	0.031*	
H27B	0.1032	1.2999	0.0768	0.031*	
C28	0.19323 (18)	1.17667 (12)	0.07203 (9)	0.0275 (4)	
C29	0.31774 (19)	1.20072 (14)	0.06228 (10)	0.0341 (4)	
H29	0.3443	1.2605	0.0737	0.041*	
C30	0.4044 (2)	1.13836 (18)	0.03599 (11)	0.0459 (5)	
H30	0.4901	1.1552	0.0303	0.055*	
C31	0.3659 (2)	1.05186 (16)	0.01803 (11)	0.0469 (6)	
H31	0.4250	1.0094	−0.0006	0.056*	
C32	0.2418 (2)	1.02721 (14)	0.02720 (10)	0.0416 (5)	
H32	0.2151	0.9677	0.0152	0.050*	
C33	0.1566 (2)	1.08942 (13)	0.05394 (9)	0.0328 (4)	
H33	0.0711	1.0721	0.0601	0.039*	

### Atomic displacement parameters (Å^2^)

**Table:**

	*U*^11^	*U*^22^	*U*^33^	*U*^12^	*U*^13^	*U*^23^
O1	0.0231 (6)	0.0249 (6)	0.0253 (6)	−0.0010 (5)	0.0020 (5)	−0.0041 (5)
O2	0.0264 (6)	0.0355 (7)	0.0285 (6)	0.0043 (5)	−0.0009 (5)	−0.0052 (5)
O3	0.0393 (7)	0.0267 (7)	0.0380 (7)	−0.0044 (6)	0.0108 (6)	−0.0073 (5)
O4	0.0549 (8)	0.0342 (7)	0.0240 (6)	−0.0080 (6)	0.0032 (6)	−0.0060 (5)
O5	0.0231 (6)	0.0207 (6)	0.0254 (6)	0.0004 (4)	−0.0005 (4)	−0.0017 (5)
O6	0.0350 (7)	0.0229 (6)	0.0292 (6)	0.0004 (5)	−0.0051 (5)	−0.0043 (5)
O7	0.0315 (7)	0.0298 (6)	0.0338 (6)	0.0024 (5)	0.0089 (6)	0.0043 (5)
O8	0.0316 (7)	0.0207 (6)	0.0420 (7)	0.0030 (5)	0.0059 (5)	0.0029 (5)
N1	0.0251 (7)	0.0242 (7)	0.0230 (7)	−0.0002 (6)	0.0005 (5)	−0.0011 (6)
N2	0.0285 (7)	0.0196 (7)	0.0230 (7)	0.0009 (6)	−0.0019 (5)	−0.0012 (6)
C1	0.0339 (10)	0.0427 (10)	0.0348 (10)	−0.0041 (9)	−0.0032 (8)	−0.0030 (9)
C2	0.0249 (9)	0.0286 (9)	0.0358 (9)	0.0007 (7)	−0.0038 (7)	0.0017 (7)
C3	0.0225 (8)	0.0206 (7)	0.0210 (8)	−0.0006 (6)	0.0013 (6)	0.0008 (6)
C4	0.0231 (8)	0.0194 (7)	0.0235 (8)	−0.0003 (6)	0.0022 (6)	0.0002 (6)
C5	0.0275 (8)	0.0199 (7)	0.0268 (8)	0.0020 (6)	0.0048 (7)	0.0036 (7)
C6	0.0302 (9)	0.0404 (10)	0.0253 (8)	0.0043 (8)	−0.0007 (7)	0.0044 (8)
C7	0.0309 (9)	0.0382 (10)	0.0230 (8)	−0.0071 (8)	−0.0012 (7)	−0.0032 (8)
C8	0.0264 (8)	0.0178 (7)	0.0212 (7)	−0.0012 (6)	0.0015 (7)	0.0006 (6)
C9	0.0252 (8)	0.0189 (8)	0.0208 (7)	0.0014 (6)	0.0015 (6)	0.0006 (6)
C10	0.0273 (8)	0.0210 (8)	0.0211 (7)	0.0014 (7)	0.0054 (6)	0.0030 (6)
C11	0.0335 (10)	0.0322 (9)	0.0286 (9)	0.0016 (8)	−0.0060 (7)	0.0049 (7)
C12	0.0273 (9)	0.0207 (8)	0.0288 (9)	0.0000 (7)	0.0031 (7)	−0.0011 (7)
C13	0.0260 (8)	0.0183 (7)	0.0266 (8)	−0.0006 (7)	0.0011 (7)	−0.0045 (6)
C14	0.0277 (9)	0.0260 (8)	0.0236 (8)	−0.0016 (7)	0.0016 (7)	−0.0015 (7)
C15	0.0262 (9)	0.0264 (9)	0.0303 (9)	−0.0012 (7)	0.0051 (7)	−0.0040 (7)
C16	0.0276 (9)	0.0297 (9)	0.0294 (9)	−0.0013 (7)	0.0024 (7)	−0.0010 (7)
C17	0.0239 (8)	0.0258 (8)	0.0297 (8)	−0.0047 (7)	0.0052 (7)	0.0008 (7)
C18	0.0270 (9)	0.0276 (9)	0.0354 (9)	−0.0016 (7)	0.0005 (7)	0.0052 (8)
C19	0.0298 (9)	0.0365 (10)	0.0306 (9)	−0.0045 (8)	−0.0027 (8)	−0.0002 (8)
C20	0.0282 (9)	0.0268 (9)	0.0436 (10)	−0.0066 (7)	0.0046 (8)	−0.0066 (8)
C21	0.0360 (10)	0.0244 (9)	0.0512 (12)	−0.0021 (8)	−0.0058 (9)	0.0059 (8)
C22	0.0405 (11)	0.0323 (10)	0.0343 (9)	−0.0064 (8)	−0.0089 (8)	0.0053 (8)
C23	0.0278 (8)	0.0246 (8)	0.0204 (7)	0.0030 (7)	0.0012 (7)	0.0004 (7)
C24	0.0197 (7)	0.0233 (8)	0.0228 (7)	0.0033 (6)	0.0025 (6)	−0.0026 (7)
C25	0.0245 (8)	0.0210 (8)	0.0249 (8)	0.0003 (6)	−0.0002 (7)	0.0010 (7)
C26	0.0265 (8)	0.0237 (8)	0.0202 (7)	0.0001 (7)	−0.0021 (6)	0.0010 (6)
C27	0.0274 (8)	0.0288 (8)	0.0225 (8)	0.0015 (7)	−0.0007 (7)	0.0032 (7)
C28	0.0344 (9)	0.0306 (9)	0.0177 (7)	0.0047 (8)	0.0015 (6)	0.0044 (7)
C29	0.0338 (10)	0.0417 (11)	0.0268 (9)	−0.0012 (8)	0.0027 (7)	0.0031 (8)
C30	0.0336 (10)	0.0732 (16)	0.0308 (10)	0.0105 (11)	0.0078 (8)	0.0135 (10)
C31	0.0612 (15)	0.0535 (13)	0.0259 (9)	0.0258 (11)	0.0061 (9)	0.0050 (9)
C32	0.0649 (14)	0.0374 (11)	0.0225 (8)	0.0111 (10)	−0.0015 (9)	−0.0019 (8)
C33	0.0441 (11)	0.0332 (10)	0.0210 (8)	0.0015 (8)	−0.0001 (7)	0.0007 (7)

### Geometric parameters (Å, °)

**Table:**

O1—C4	1.434 (2)	C10—H10	1.0000
O1—H1O	0.961 (10)	C11—H11	1.0000
O2—C13	1.239 (2)	C12—C13	1.514 (2)
O3—C15	1.202 (2)	C12—H12A	0.9900
O4—C15	1.333 (2)	C12—H12B	0.9900
O4—H4O	0.961 (10)	C14—C15	1.526 (2)
O5—C9	1.436 (2)	C14—C16	1.539 (2)
O5—H5O	0.949 (10)	C14—H14	1.0000
O6—C24	1.230 (2)	C16—C17	1.514 (2)
O7—C26	1.202 (2)	C16—H16A	0.9900
O8—C26	1.316 (2)	C16—H16B	0.9900
O8—H8O	0.968 (10)	C17—C22	1.384 (3)
N1—C13	1.332 (2)	C17—C18	1.390 (3)
N1—C14	1.447 (2)	C18—C19	1.388 (3)
N1—H1N	0.950 (10)	C18—H18	0.9500
N2—C24	1.337 (2)	C19—C20	1.378 (3)
N2—C25	1.447 (2)	C19—H19	0.9500
N2—H2N	0.956 (10)	C20—C21	1.377 (3)
C1—C7	1.499 (3)	C20—H20	0.9500
C1—C2	1.511 (3)	C21—C22	1.391 (3)
C1—H1A	0.9900	C21—H21	0.9500
C1—H1B	0.9900	C22—H22	0.9500
C2—C6	1.541 (3)	C23—C24	1.522 (2)
C2—C3	1.542 (2)	C23—H23A	0.9900
C2—H2	1.0000	C23—H23B	0.9900
C3—C4	1.541 (2)	C25—C26	1.522 (2)
C3—C8	1.589 (2)	C25—C27	1.539 (2)
C3—H3	1.0000	C25—H25	1.0000
C4—C5	1.529 (2)	C27—C28	1.509 (2)
C4—C12	1.538 (2)	C27—H27A	0.9900
C5—C6	1.561 (3)	C27—H27B	0.9900
C5—C10	1.579 (2)	C28—C29	1.381 (3)
C5—H5	1.0000	C28—C33	1.387 (3)
C6—C11	1.616 (3)	C29—C30	1.388 (3)
C6—H6	1.0000	C29—H29	0.9500
C7—C11	1.537 (3)	C30—C31	1.381 (4)
C7—C8	1.549 (2)	C30—H30	0.9500
C7—H7	1.0000	C31—C32	1.378 (4)
C8—C9	1.553 (2)	C31—H31	0.9500
C8—H8	1.0000	C32—C33	1.380 (3)
C9—C10	1.533 (2)	C32—H32	0.9500
C9—C23	1.541 (2)	C33—H33	0.9500
C10—C11	1.555 (3)		
			
C4—O1—H1O	100.1 (15)	C4—C12—H12B	109.6
C15—O4—H4O	108.7 (16)	H12A—C12—H12B	108.1
C9—O5—H5O	109.6 (17)	O2—C13—N1	122.52 (16)
C26—O8—H8O	108.7 (18)	O2—C13—C12	120.59 (15)
C13—N1—C14	120.67 (14)	N1—C13—C12	116.83 (15)
C13—N1—H1N	120.4 (15)	N1—C14—C15	111.29 (14)
C14—N1—H1N	118.9 (15)	N1—C14—C16	110.04 (14)
C24—N2—C25	120.00 (14)	C15—C14—C16	112.97 (14)
C24—N2—H2N	117.4 (15)	N1—C14—H14	107.4
C25—N2—H2N	122.3 (15)	C15—C14—H14	107.4
C7—C1—C2	93.63 (15)	C16—C14—H14	107.4
C7—C1—H1A	113.0	O3—C15—O4	125.04 (17)
C2—C1—H1A	113.0	O3—C15—C14	125.29 (16)
C7—C1—H1B	113.0	O4—C15—C14	109.65 (14)
C2—C1—H1B	113.0	C17—C16—C14	110.13 (14)
H1A—C1—H1B	110.4	C17—C16—H16A	109.6
C1—C2—C6	106.67 (16)	C14—C16—H16A	109.6
C1—C2—C3	105.01 (15)	C17—C16—H16B	109.6
C6—C2—C3	100.90 (14)	C14—C16—H16B	109.6
C1—C2—H2	114.3	H16A—C16—H16B	108.1
C6—C2—H2	114.3	C22—C17—C18	118.22 (17)
C3—C2—H2	114.3	C22—C17—C16	120.73 (16)
C4—C3—C2	101.98 (13)	C18—C17—C16	120.96 (16)
C4—C3—C8	114.03 (13)	C19—C18—C17	120.83 (17)
C2—C3—C8	101.45 (13)	C19—C18—H18	119.6
C4—C3—H3	112.8	C17—C18—H18	119.6
C2—C3—H3	112.8	C20—C19—C18	120.12 (17)
C8—C3—H3	112.8	C20—C19—H19	119.9
O1—C4—C5	113.61 (13)	C18—C19—H19	119.9
O1—C4—C12	105.98 (13)	C21—C20—C19	119.85 (18)
C5—C4—C12	110.02 (13)	C21—C20—H20	120.1
O1—C4—C3	114.11 (13)	C19—C20—H20	120.1
C5—C4—C3	100.11 (13)	C20—C21—C22	119.90 (18)
C12—C4—C3	113.13 (13)	C20—C21—H21	120.1
C4—C5—C6	102.18 (13)	C22—C21—H21	120.1
C4—C5—C10	116.27 (13)	C17—C22—C21	121.07 (17)
C6—C5—C10	90.56 (13)	C17—C22—H22	119.5
C4—C5—H5	114.9	C21—C22—H22	119.5
C6—C5—H5	114.9	C24—C23—C9	114.38 (13)
C10—C5—H5	114.9	C24—C23—H23A	108.7
C2—C6—C5	108.05 (14)	C9—C23—H23A	108.7
C2—C6—C11	100.85 (15)	C24—C23—H23B	108.7
C5—C6—C11	89.21 (13)	C9—C23—H23B	108.7
C2—C6—H6	118.0	H23A—C23—H23B	107.6
C5—C6—H6	118.0	O6—C24—N2	120.59 (15)
C11—C6—H6	118.0	O6—C24—C23	121.95 (14)
C1—C7—C11	106.77 (16)	N2—C24—C23	117.47 (14)
C1—C7—C8	105.12 (15)	N2—C25—C26	110.36 (13)
C11—C7—C8	101.62 (14)	N2—C25—C27	110.76 (13)
C1—C7—H7	114.1	C26—C25—C27	108.83 (13)
C11—C7—H7	114.1	N2—C25—H25	109.0
C8—C7—H7	114.1	C26—C25—H25	109.0
C7—C8—C9	100.84 (13)	C27—C25—H25	109.0
C7—C8—C3	101.14 (13)	O7—C26—O8	124.36 (16)
C9—C8—C3	115.70 (13)	O7—C26—C25	123.14 (15)
C7—C8—H8	112.6	O8—C26—C25	112.42 (14)
C9—C8—H8	112.6	C28—C27—C25	112.28 (14)
C3—C8—H8	112.6	C28—C27—H27A	109.1
O5—C9—C10	114.79 (13)	C25—C27—H27A	109.1
O5—C9—C23	103.32 (13)	C28—C27—H27B	109.1
C10—C9—C23	110.87 (13)	C25—C27—H27B	109.1
O5—C9—C8	116.65 (13)	H27A—C27—H27B	107.9
C10—C9—C8	99.67 (13)	C29—C28—C33	118.50 (18)
C23—C9—C8	111.85 (13)	C29—C28—C27	120.95 (17)
C9—C10—C11	103.12 (13)	C33—C28—C27	120.50 (17)
C9—C10—C5	114.46 (13)	C28—C29—C30	120.6 (2)
C11—C10—C5	90.82 (13)	C28—C29—H29	119.7
C9—C10—H10	115.1	C30—C29—H29	119.7
C11—C10—H10	115.1	C31—C30—C29	120.1 (2)
C5—C10—H10	115.1	C31—C30—H30	120.0
C7—C11—C10	107.37 (14)	C29—C30—H30	120.0
C7—C11—C6	100.80 (14)	C32—C31—C30	119.9 (2)
C10—C11—C6	89.41 (13)	C32—C31—H31	120.1
C7—C11—H11	118.2	C30—C31—H31	120.1
C10—C11—H11	118.2	C31—C32—C33	119.7 (2)
C6—C11—H11	118.2	C31—C32—H32	120.2
C13—C12—C4	110.24 (13)	C33—C32—H32	120.2
C13—C12—H12A	109.6	C32—C33—C28	121.3 (2)
C4—C12—H12A	109.6	C32—C33—H33	119.3
C13—C12—H12B	109.6	C28—C33—H33	119.3
			
C7—C1—C2—C6	−52.22 (17)	C5—C10—C11—C7	100.82 (14)
C7—C1—C2—C3	54.33 (17)	C9—C10—C11—C6	−115.68 (13)
C1—C2—C3—C4	−152.25 (15)	C5—C10—C11—C6	−0.31 (12)
C6—C2—C3—C4	−41.51 (16)	C2—C6—C11—C7	0.97 (16)
C1—C2—C3—C8	−34.36 (17)	C5—C6—C11—C7	−107.27 (14)
C6—C2—C3—C8	76.38 (15)	C2—C6—C11—C10	108.55 (14)
C2—C3—C4—O1	174.09 (13)	C5—C6—C11—C10	0.31 (12)
C8—C3—C4—O1	65.62 (17)	O1—C4—C12—C13	64.48 (16)
C2—C3—C4—C5	52.38 (15)	C5—C4—C12—C13	−172.31 (14)
C8—C3—C4—C5	−56.08 (16)	C3—C4—C12—C13	−61.28 (18)
C2—C3—C4—C12	−64.64 (17)	C14—N1—C13—O2	2.7 (2)
C8—C3—C4—C12	−173.11 (13)	C14—N1—C13—C12	−174.58 (14)
O1—C4—C5—C6	−162.75 (13)	C4—C12—C13—O2	−51.7 (2)
C12—C4—C5—C6	78.63 (16)	C4—C12—C13—N1	125.61 (16)
C3—C4—C5—C6	−40.69 (15)	C13—N1—C14—C15	−79.26 (19)
O1—C4—C5—C10	−65.99 (18)	C13—N1—C14—C16	154.73 (15)
C12—C4—C5—C10	175.39 (14)	N1—C14—C15—O3	−10.9 (3)
C3—C4—C5—C10	56.07 (17)	C16—C14—C15—O3	113.5 (2)
C1—C2—C6—C5	125.34 (16)	N1—C14—C15—O4	167.77 (15)
C3—C2—C6—C5	15.89 (18)	C16—C14—C15—O4	−67.9 (2)
C1—C2—C6—C11	32.60 (17)	N1—C14—C16—C17	−64.59 (18)
C3—C2—C6—C11	−76.85 (16)	C15—C14—C16—C17	170.36 (14)
C4—C5—C6—C2	15.59 (17)	C14—C16—C17—C22	−74.1 (2)
C10—C5—C6—C2	−101.47 (15)	C14—C16—C17—C18	102.51 (18)
C4—C5—C6—C11	116.75 (13)	C22—C17—C18—C19	1.8 (3)
C10—C5—C6—C11	−0.31 (12)	C16—C17—C18—C19	−174.88 (17)
C2—C1—C7—C11	53.08 (17)	C17—C18—C19—C20	−1.0 (3)
C2—C1—C7—C8	−54.34 (17)	C18—C19—C20—C21	−0.3 (3)
C1—C7—C8—C9	153.91 (15)	C19—C20—C21—C22	0.6 (3)
C11—C7—C8—C9	42.77 (16)	C18—C17—C22—C21	−1.5 (3)
C1—C7—C8—C3	34.72 (17)	C16—C17—C22—C21	175.23 (17)
C11—C7—C8—C3	−76.42 (15)	C20—C21—C22—C17	0.3 (3)
C4—C3—C8—C7	108.78 (15)	O5—C9—C23—C24	−71.60 (17)
C2—C3—C8—C7	−0.01 (15)	C10—C9—C23—C24	164.95 (13)
C4—C3—C8—C9	0.87 (19)	C8—C9—C23—C24	54.67 (18)
C2—C3—C8—C9	−107.93 (15)	C25—N2—C24—O6	3.3 (2)
C7—C8—C9—O5	−176.46 (13)	C25—N2—C24—C23	−176.58 (14)
C3—C8—C9—O5	−68.37 (18)	C9—C23—C24—O6	−136.58 (16)
C7—C8—C9—C10	−52.31 (15)	C9—C23—C24—N2	43.3 (2)
C3—C8—C9—C10	55.78 (16)	C24—N2—C25—C26	−73.50 (18)
C7—C8—C9—C23	64.93 (16)	C24—N2—C25—C27	165.92 (14)
C3—C8—C9—C23	173.02 (13)	N2—C25—C26—O7	−25.6 (2)
O5—C9—C10—C11	165.98 (13)	C27—C25—C26—O7	96.15 (19)
C23—C9—C10—C11	−77.43 (16)	N2—C25—C26—O8	157.55 (14)
C8—C9—C10—C11	40.54 (14)	C27—C25—C26—O8	−80.72 (17)
O5—C9—C10—C5	68.97 (18)	N2—C25—C27—C28	−51.65 (19)
C23—C9—C10—C5	−174.44 (14)	C26—C25—C27—C28	−173.14 (14)
C8—C9—C10—C5	−56.47 (16)	C25—C27—C28—C29	−68.3 (2)
C4—C5—C10—C9	1.2 (2)	C25—C27—C28—C33	109.20 (18)
C6—C5—C10—C9	105.14 (15)	C33—C28—C29—C30	−0.9 (3)
C4—C5—C10—C11	−103.58 (15)	C27—C28—C29—C30	176.60 (16)
C6—C5—C10—C11	0.32 (13)	C28—C29—C30—C31	1.2 (3)
C1—C7—C11—C10	−127.46 (15)	C29—C30—C31—C32	−0.9 (3)
C8—C7—C11—C10	−17.57 (18)	C30—C31—C32—C33	0.3 (3)
C1—C7—C11—C6	−34.67 (17)	C31—C32—C33—C28	−0.1 (3)
C8—C7—C11—C6	75.21 (16)	C29—C28—C33—C32	0.4 (3)
C9—C10—C11—C7	−14.54 (17)	C27—C28—C33—C32	−177.14 (16)

### Hydrogen-bond geometry (Å, °)

**Table:**

*D*—H···*A*	*D*—H	H···*A*	*D*···*A*	*D*—H···*A*
N1—H1N···O6^i^	0.95 (1)	1.90 (1)	2.8399 (18)	172 (2)
N2—H2N···O5	0.96 (1)	2.01 (2)	2.7579 (18)	134 (2)
O1—H1O···O2	0.96 (1)	1.79 (2)	2.6649 (16)	150 (2)
O5—H5O···O1	0.95 (1)	1.58 (1)	2.4886 (16)	158 (3)
O4—H4O···O7^ii^	0.96 (1)	1.84 (2)	2.7425 (18)	154 (2)
O8—H8O···O5^iii^	0.97 (1)	1.68 (1)	2.6501 (17)	177 (3)

Symmetry codes: (i) −*x*+1, *y*−1/2, −*z*+1/2; (ii) −*x*+1/2, −*y*+2, *z*−1/2; (iii) −*x*, *y*+1/2, −*z*+1/2.
